# Airflow analysis of Pyeongtaek St Mary's Hospital during hospitalization of the first Middle East respiratory syndrome patient in Korea

**DOI:** 10.1098/rsos.181164

**Published:** 2019-03-13

**Authors:** Seongmin Jo, Jinkwan Hong, Sang-Eun Lee, Moran Ki, Bo Youl Choi, Minki Sung

**Affiliations:** 1 Department of Architectural Engineering, Sejong University, Seoul, South Korea; 2 Department of HVAC and Firefighting Engineering, Gachon University, Gyeonggi-do, South Korea; 3 Division of Vectors and Parasitic Diseases, Korea Centers for Disease Control and Prevention, Cheongju, South Korea; 4 Department of Cancer Control and Population Health, Graduate School of Cancer Science and Policy, National Cancer Center, Goyang, South Korea; 5 Department of Preventive Medicine, Hanyang University Medical College, Seoul, South Korea

**Keywords:** Middle East respiratory syndrome, hospital infection, airflow analysis, transmission route, computational fluid dynamics, ventilation

## Abstract

Middle East respiratory syndrome (MERS) is known to be transmitted through close contact. However, epidemiological surveys of MERS in Korea indicated that some secondary patients were infected without close contact. Therefore, the possibility of other transmission routes must be identified. In this study, the possibility of MERS spreading through airflow was investigated on the eighth floor of Pyeongtaek St Mary's Hospital. Computational fluid dynamics was used to analyse the indoor airflow and passive tracer diffusion during the index patient's stay. Six cases were simulated for different outdoor wind directions and indoor mechanical ventilation operations. When a passive tracer was released in ward 8104, where the index patient was hospitalized, the passive tracer spread through the indoor airflow, which was created by the outdoor airflow. Ward 8109, which had the largest number of infected cases and was far distant from ward 8104, showed passive tracer concentration in all cases. This result indicates that MERS may have spread through airflow. The study results do not imply that the infection pathway of MERS is airborne. However, the results show the possibility of MERS spreading through airflow in specific environments such as poor ventilation environments.

## 1. Introduction

Middle East respiratory syndrome (MERS) was first reported in Saudi Arabia in 2012 and is a severe respiratory disease that causes fever, cough and respiratory disturbance, as in the case of severe acute respiratory syndrome (SARS) [1,2]. The reported cases of MERS infection increased in the Middle East in May 2014, and since then, there has been a growing concern about MERS infection [3]. In May 2015, the first case of MERS infection was reported in Korea; the patient had contracted the disease while visiting the Arabian Peninsula and had returned to Korea, thereby spreading MERS in Korea [4]. According to the US Centers for Disease Control and Prevention (CDC), the region with the highest number of infected cases outside the Arabian Peninsula is Korea [5]. MERS spread in Korea from May 2015 to July 2015; in less than two months, 186 people were infected and 38 people died. The Korea CDC reported that MERS rapidly spread over a short period because of hospital infection. According to epidemiological findings, 87.6% of the infected patients were infected in the hospital [6]. Similarly, in the MERS cases reported in Jeddah in 2014, infection was found to spread in the hospital through super-spreading events [7]. The MERS events in Korea and Jeddah indicate that MERS infection can gradually increase when a dense population is confined to relatively small spaces such as hospitals.

The index patient was admitted to a ward on the eighth floor of Pyeongtaek St Mary's Hospital on 15–17 May 2015, with fever and muscle pain [8]. In addition to the index patient, 30 people (15 inpatients, 13 family members and 2 nurses) were infected on the eighth floor of the hospital [9]. To confirm the cause of the infection of secondary patients at the hospital, an epidemiological investigation of the infection transmission route was conducted. The investigation results showed that 23% of the infected patients did not come in close contact with other infected patients [10]. Similar to SARS, MERS is known to be an infection caused by close contact [11]. However, the infection of a secondary patient with no close contact with the index patient does not support the claim that MERS is spread only through close contact. Therefore, the possibility of other infection transmission routes of MERS, such as air transmission, is being studied [12,13]. In the case of SARS, the airflow in the Prince of Wales Hospital was analysed, where the first SARS-infected patient was hospitalized. The results showed that the pattern of the infection of the secondary patient was similar to the indoor airflow pattern [14]. When SARS was reported in Toronto, Canada, a field experiment was performed to determine the infection transmission route, and the results indicated that SARS could possibly be an opportunistic airborne infection [15]. A new risk calculation method was applied to confirm the spatial risk distribution of SARS, and the results showed that SARS was more likely to be transmitted through the air [16].

According to Oh *et al.*'s review [17] on MERS, to prevent the recirculation of pathogen-bearing droplets, the air changes per hour (ACH) should be over 6, and to prevent a high concentration of infectious droplets, proper ventilation is mandatory. However, the index patient was admitted to a ward with no supply and exhaust vent. The ward was originally a seven-person ward and was separated into two two-person wards. In the process of separating the wards, vents were installed in only one of the wards. Because of this construction lapse, the index patient's ward was not mechanically ventilated, and only natural ventilation was provided through the window. It is possible that the droplets produced by coughing and sneezing by the index patient accumulated in the ward at a high concentration due to lack of ventilation. MERS bacteria are known to survive for up to 72 h on plastic or steel surfaces at a room temperature of 20°C and a humidity of 40% [18], and droplets are known to shrink their size during the evaporation process [19,20]. A person who infects more than five people is called a super-spreader, and the index patient was also surveyed as a super-spreader [21]. The results of previous studies indicate that the droplets from the index patient, who was a super-spreader, can partially evaporate and become small particles such as droplet nuclei and then spread to the adjacent wards through airflow [14,22,23].

SARS is known to cause super-spreading events through super-spreaders [24,25]. Considering that the first MERS-infected patient in Korea was a super-spreader and that the ward in which the index patient was hospitalized was not properly ventilated, it is necessary to review the possibility of distant propagation through airflow. The purpose of this study was to investigate the possibility of distant propagation through the airflow on the eighth floor of Pyeongtaek St Mary's Hospital, where the index patient was hospitalized. Computational fluid dynamics (CFD) was used to simulate the situation during the period of the index patient's hospitalization and observe the possibility of propagation through airflow.

## 2. Material and methods

### 2.1. Epidemiological survey of hospital

**Figure 1.**
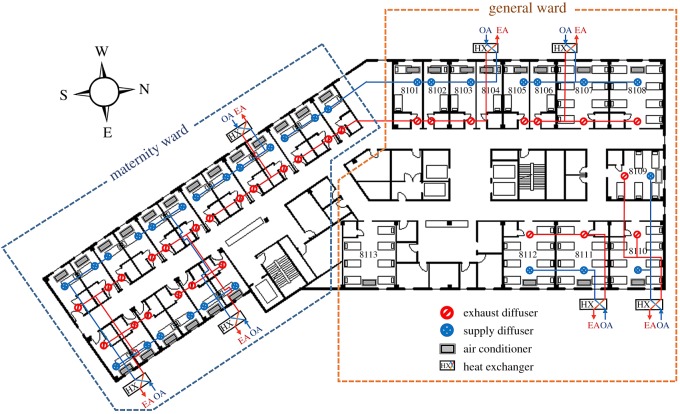
Layout and mechanical ventilation system of Pyeongtaek St Mary's hospital 8th floor.

Pyeongtaek St Mary's Hospital is a nine-storey building completely built in February 2015. The medical department is located from the first floor to the third floor, and the ward department is located from the fourth floor to the ninth floor. The eighth floor, where the index patient was hospitalized, is divided into a maternity ward department, which consists of a one-person ward, and a general ward department, which consists of a two-person ward and seven-person ward (figure 1). The room air conditioner and radiator installed in each ward control the cooling and heating of the ward. The ventilation of the ward is controlled by a sensible heat exchanger installed in groups of two to six wards; air handling unit was not installed. Awning windows are present in each ward and can be opened or closed by the patient. In the corridor, ventilation is not provided and only a ceiling-type air conditioner is installed. The air conditioner and ventilation system of each ward can be controlled individually, so the occupant can freely manipulate them. According to interviews with patients who were present during the index patient's hospitalization, they used the air conditioner according to their needs; however, most of them did not recognize the ventilation devices, making it difficult to determine whether the air conditioner and ventilation system were in operation. In the case of ward 8104, the index patient stated that the air conditioner was not in use except for approximately half a day and that the window was kept open for ventilation.

The maximum airflow rate was designed as 200 cubic metres per hour (CMH) for a one-person ward, 250 CMH for a two-person ward and 500 CMH for a seven-person ward, and the ACH was approximately 5. However, the actual air supply and exhaust airflow rates during the period of admission of the index patient were not confirmed because of the adjustment of the ventilation system between the closure (29 May 2015) and reopening (6 July 2015) of the hospital.

**Figure 2.**
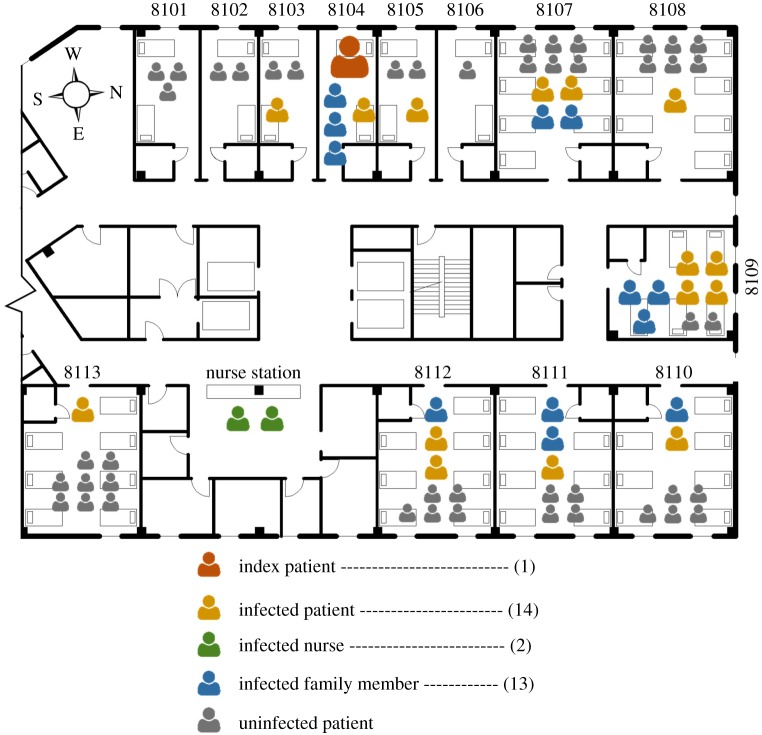
Distribution of infected patients on the eighth floor.

Kim *et al*. [26] analysed the epidemiological features of the index patient and secondary patient at Pyeongtaek St Mary's Hospital. According to this study, the index patient was admitted to the two-person ward (ward 8104) from 15 May to 17 May 2015, due to fever and coughing symptoms. Figure 2 shows the patients, visiting family members and nurses who were infected on the eighth floor. In ward 8104, one patient using the same room and three visiting family members were infected. In the adjoining and close-distance wards to ward 8104, i.e. wards 8103 and 8105, one patient was infected. Infected patients were also confirmed in distant wards. At least one infected case occurred in all east-facing wards. Ward 8109 had the highest number of confirmed infected cases, with four patients and three visiting family members being infected. Absolute confirmation of close contact between the index patient, secondary patient, visiting family members and nurse was impossible.

Sung *et al*. [22] performed a diffusion experiment using aerosol and tracer gas on 8 May 2015, when the hospital was shut down. He confirmed the possibility of diffusion of infectious bacteria through the indoor airflow. However, because of the limitations of the field experiment, a detailed analysis was not carried out. Therefore, this study reproduced and analysed the phenomenon observed by Sung *et al*. We used CFD to analyse the indoor airflow and passive tracer diffusion path on the eighth floor, where the index patient was hospitalized.

The eighth-floor ward can be naturally ventilated through the windows, so it was necessary to check the outdoor and indoor airflow conditions. Therefore, the facade and the eighth floor of Pyeongtaek St Mary's Hospital were modelled and simulated to observe both outdoor and indoor airflow conditions.

### 2.2. Computational fluid dynamics analysis

**Figure 3.**
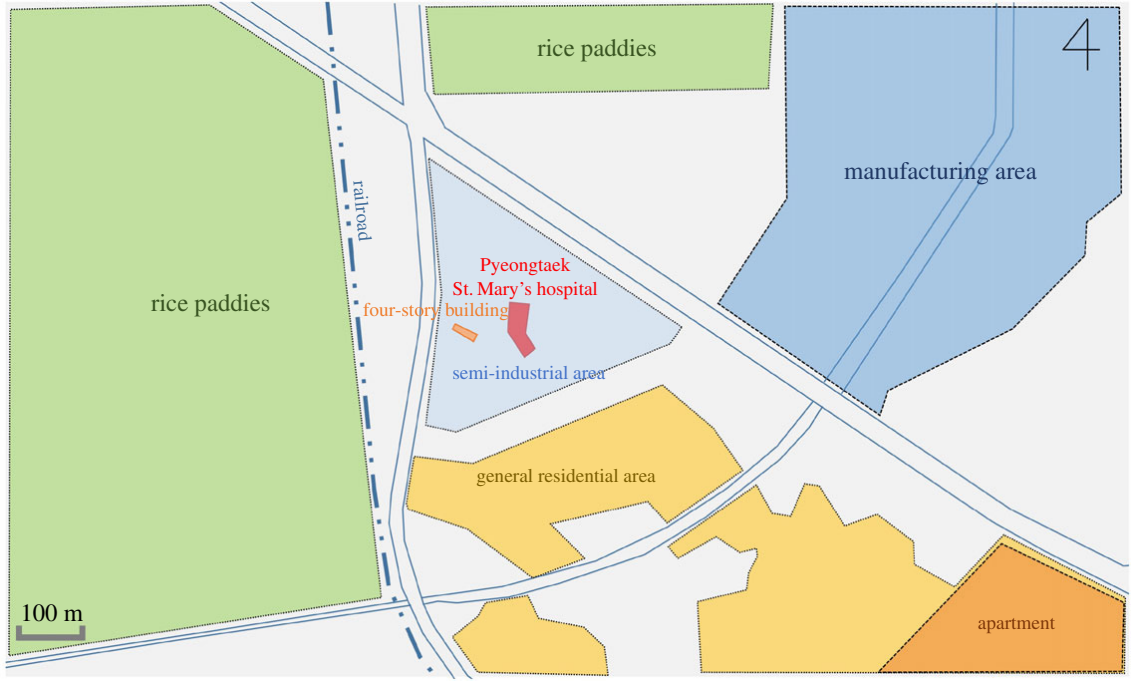
Terrain conditions near the hospital.

Pyeongtaek St Mary's Hospital is tilted approximately 5° eastward from the north direction. A survey of the surrounding terrain conditions (figure 3) indicates that a housing area is located towards the south, a factory area towards the northeast and rice paddies towards the west and north. No high-rise buildings with over five storeys are present within 500 m. However, a relatively high four-storey building is located approximately 40 m to the west of the hospital, so the building was included in the simulation domain.

**Figure 4.**
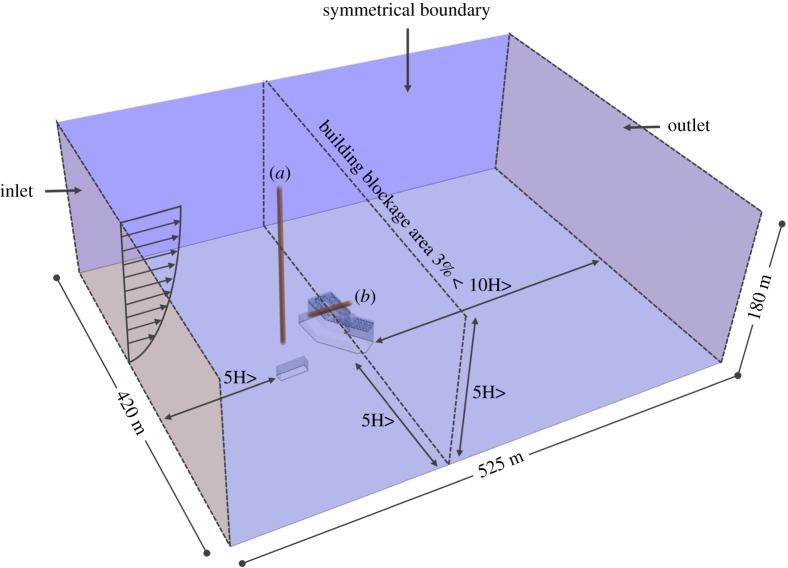
Domain for CFD simulation.

**Figure 5.**
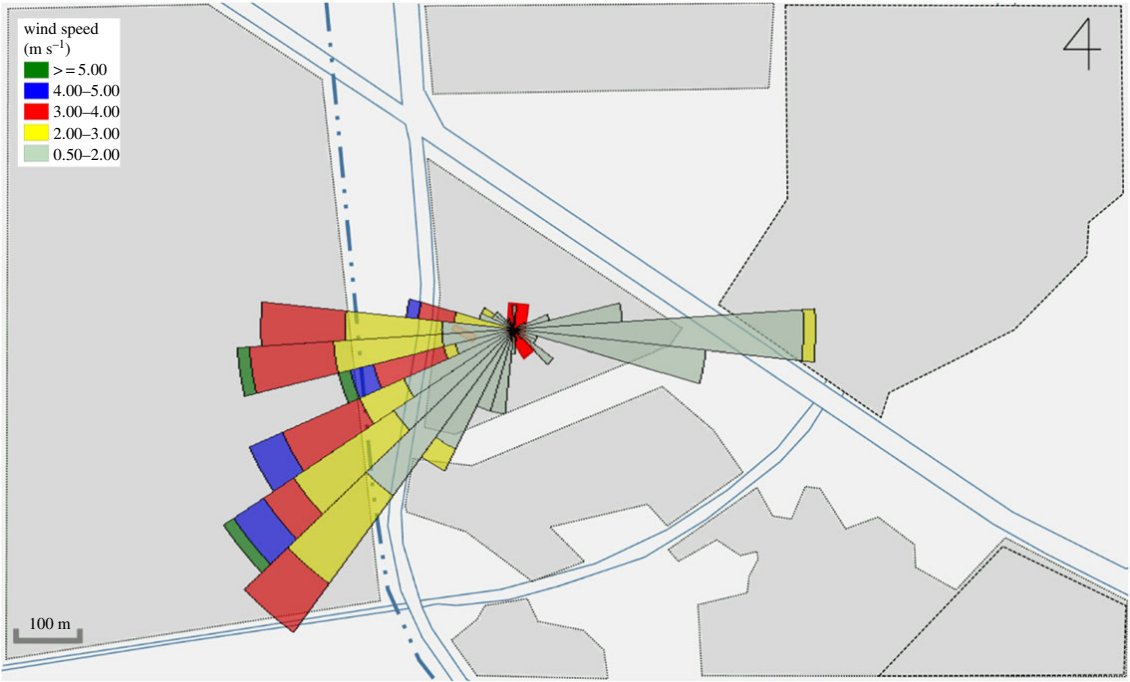
Local wind profile for 15–17 May 2015.

CFD analysis was performed using STAR-CCM+, a commercial simulation program. The size of the domain used in the analysis was modelled to be 420 × 525 × 180 m (figure 4). There was sufficient space between the building and analysis domain to allow the airflow to fully develop and hence obtain reliable analysis results [27]. The analytical mesh was a trimmer mesh, and the prism layer mesh was applied to the wall. Mesh independence test was performed and compared with coarse (3 million), medium (6 million) and fine mesh size (9 million) for prediction accuracy of the CFD simulation results [28]. Outdoor velocity data were extracted at the front of the hospital (figure 4*a*) at constant height of 1 m and indoor velocity data were extracted from window height (figure 4*b*). Realizable *k*–*ɛ* turbulence model [29,30] was used as the turbulence model. As a boundary condition, the airflow was set in the windward direction according to the wind speed profile and it was set to escape to the leeward direction due to pressure formation [31]. The side and top parts of the domain were set to be symmetrical. Figure 5 shows the wind speed and direction when the index patient was hospitalized, i.e. from noon on 15 May 2015 to the morning of 17 May 2015. Wind data at the height of 10 m were obtained from the nearest weather observatory located 3.6 km away from the hospital. The CFD cases were selected according to the dominant wind directions, i.e. west, west-southwest winds. The average wind speed at the height of 10 m was 2.40 m s^−1^ for the west wind and 2.62 m s^−1^ for the west-southwest wind.

Wind blowing from outside the building is affected by the terrain of the surface and blockage of the building; the wind speed is lower at low height, and the wind speed increases with the distance from the surface. To employ these conditions in the CFD analysis, the wind speed at the height of 0–180 m was calculated using the exponential law [32] based on the average wind speed at the height of 10 m blowing from each direction. To determine the roughness of the earth's surface, the roughness value *α* should be selected. This value was chosen to be 0.22 (for regions where houses with a height of 3.5 m are concentrated or regions where middle-class buildings are scattered) according to the Korea Building Code [33].

**Figure 6.**
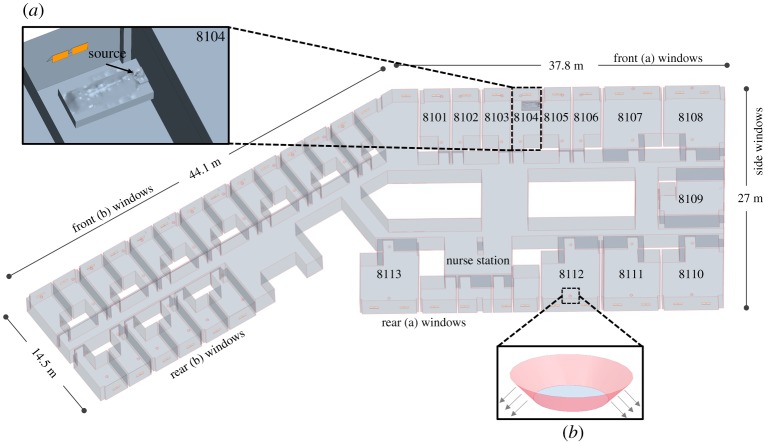
Indoor model of the eighth floor. (*a*) Ward 8104 and source location and (*b*) ventilation diffuser.

For indoor conditions, both the maternity and general ward departments were included in the CFD domain to observe the passive tracer diffusion on the eighth floor when the passive tracer was generated in ward 8104. As shown in figure 6, 50 windows were placed on the front, 36 windows on the rear and six windows on the side like the actual eighth floor of the hospital. According to the interviews with patients and medical staffs, most of the windows were open for ventilation in the daytime during the outbreak because outdoor temperature was moderate. Therefore, awning windows were opened at the angle of 30°. As shown in figure 6, the air supply and exhaust vents of each room were modelled by reflecting the shapes of the actual air supply and exhaust vents installed in the hospital. The bathroom was not modelled, and only the door undercut (exhaust) was modelled for analysis efficiency. The design airflow rate was used for the supply and exhaust air vents and bathroom exhaust.

The passive tracer was released from the mouth of a lying patient model. The model was set to exhale 5 l of passive tracer per minute, which is considered to be the average respiration rate of a person. Droplets from a patient evaporate and can rapidly turn into small particles. In this study, the airborne infectious particles were considered as gas-phase substances (refer to the previous studies [34–38]).

The CFD analysis cases (table 1) are based on the two wind directions (west, west-southwest). In case 1, mechanical ventilation system is not operated only in ward 8104 which represents the actual situation when the index patient was hospitalized. Case 1T represents non-isothermal simulation. In case 2, all the ventilation systems are not in operation. In case 3, mechanical ventilation systems are operated in all the wards, to observe improved the airflow and passive tracer concentration reduction.

**Table 1.** CFD simulation cases.

	wind direction	mechanical ventilation system	thermal condition
case W-1T	west	operated except in ward 8104 (actual situation)	non-isothermal
case W-1			
case W-2		not operated in all wards	isothermal
case W-3		operated in all wards	
case WSW-1	west southwest	operated except in ward 8104 (actual situation)	
case WSW-2		not operated in all wards	
case WSW-3		operated in all wards	

The average outside temperature in Pyeongtaek district was 17.8°C at the time of the index patient admission, so air conditioning was not required, and the survey results indicated that the air conditioner in ward 8104 was not operated most of the time. Therefore, in the simulation, air conditioning was not considered. In addition, due to the characteristics of the hospital building, where family members visit wards from time to time, medical staff frequently shift duties and the heat generation value of rooms change with time, specifying accurate heat generation value was impossible. However, non-isothermal simulation should be considered and compared with isothermal simulation. Thereby, average outdoor temperature of 17.8°C and heat generation values for patient and nurse were applied to the floor and ceiling light was applied to the ceiling for one case (caseW-1T) and was compared to isothermal simulation (caseW-1) results.

## 3. Results

**Figure 7.**
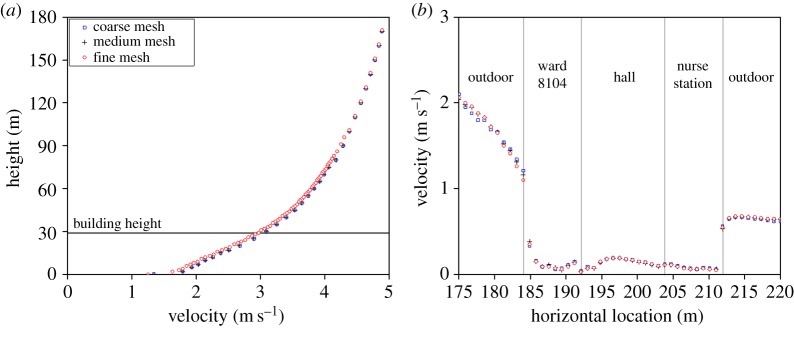
Mesh independence test results. (*a*) Outdoor mesh independence and (*b*) indoor mesh independence.

As shown in figure 7, the maximum difference of outdoor air velocity between coarse, medium and fine meshes in range of 0–180 and 0–30 m where building is located, were both 0.15 m s^−1^ which was not significantly large. However, maximum difference of indoor velocity of the three meshes showed a difference. Coarse mesh and fine showed a maximum velocity difference of 0.1 m s^−1^ and medium mesh and fine mesh showed 0.06 m s^−1^ difference. Therefore, medium mesh with 6 million cell was considered independent and was selected for the simulation.

**Figure 8.**
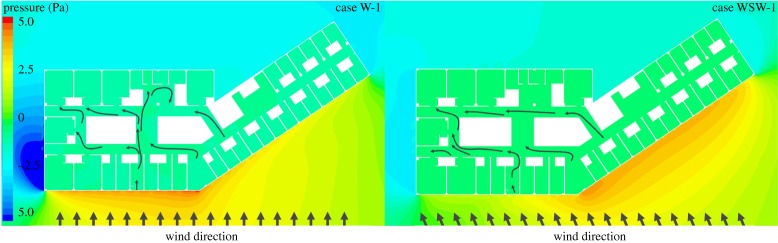
Pressure distribution of case W-1 and case WSW-1 at height of the eightth floor.

Figure 8 shows the pressure distribution at the height of the patient's mouth on the eighth floor. In the case of the wind blowing from the west, when the wind direction was more perpendicular to the surface of the building, a positive pressure (greater than 3 Pa) was formed that was higher than that formed when the wind direction was less perpendicular to the building surface. Positive pressure was also formed on the west-southwest outer surface of the building, and a relatively high positive pressure (greater than 4 Pa) was observed in the angular part of the centre of the west side of the building. When the wind was blowing from the west-southwest direction, high positive pressure (greater than 3 Pa) was created on the west-southwest outer surface of the building, and the highest positive pressure (greater than 4 Pa) was formed in the west angular part of the building, which is similar to the case in which the wind was blowing from the west. In all the cases, in the side part of the building, a negative pressure was formed and a vortex was generated making the airflow separate and reattach to the side of the building.

In the case of the west wind, air mainly flowed through front (a) window to rear (a) windows. In the case of the west-southwest wind, compared to the west wind, the airflow through the front (a) window decreased and the airflow through the front (b) window increased. The air mainly flowed in through front (b) and flowed out through the side windows and rear (a) windows.

**Figure 9.**
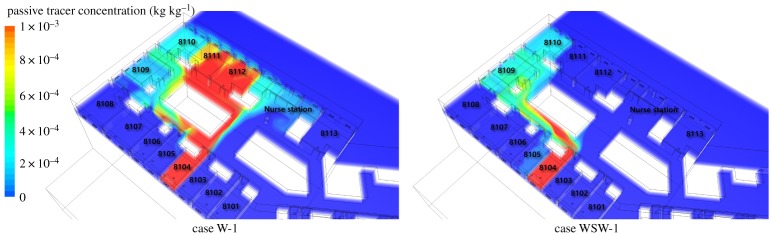
Passive tracer distribution of case W-1 and case WSW-1 in the occupied zone.

Figure 9 shows the results of the indoor passive tracer distribution. The analysis results for the west wind airflow indicated that the outdoor air mainly flowed in through the west-facing windows and flowed out through the rear windows. The passive tracer from the lying patient's mouth in ward 8104 mainly spread to wards 8111 and 8112. In the case of the west-southwest wind, compared to the west wind, the air mainly flowed in through west-southwest windows and flowed out through the side and rear windows. The passive tracer spread to ward 8109, 8110 and some portion spread to adjacent ward 8105.

**Figure 10.**
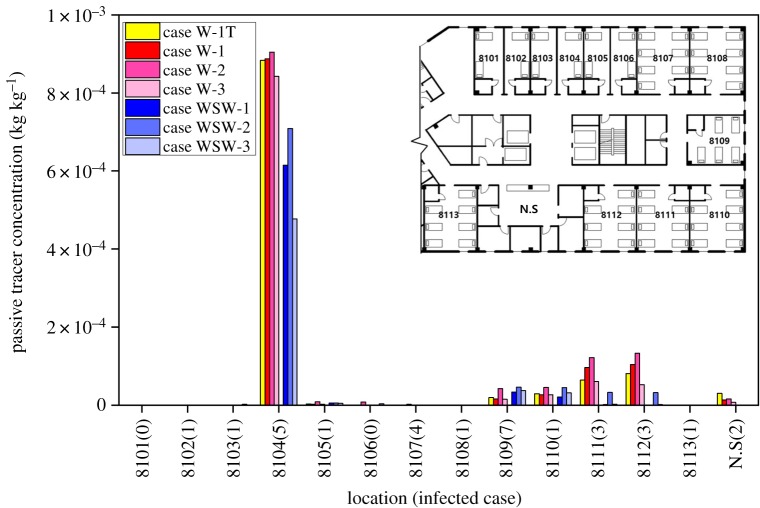
Passive tracer concentration at different wards.

Figure 10 shows the volume-averaged concentration of the passive tracer in residential area which is 0–1.8 m in each room. In all the cases, no contaminants were detected in the maternity ward department, where no MERS infection cases were reported. Among all the wards, index patient ward (8104) had the highest concentration of the passive tracer in all the cases. It is difficult to directly relate the reported infected cases with the concentration of the passive tracer in wards because the wind does not continuously blow in one direction. However, in case WSW-1, which represents the actual situation when the index patient was hospitalized and the wind was blowing from the west-southwest, ward 8109, where the highest infected cases were reported, showed the highest concentration. In the case of ward 8107, where four infected cases were reported, the highest passive tracer concentration was only 0.02% compared to ward 8014 (100%). However, in a situation where the west or west-southwest wind was blowing and the east wind was blown after the passive tracer was spread, the passive tracer could re-spread to the west-facing wards.

The concentration in each ward is shown in table 2, and the highest concentration in ward 8104 (case W-2) is considered to be 100%. In case W-1, where the wind was blowing from the west, relatively high concentration was observed in ward 8111 (10.7%) and 8112 (11.4%); both wards had three infected cases. Comparing with case W-1T, which is non-isothermal simulation, concentration difference of ward 8014 was not significant. However, wards 8111 and 8112 showed 3.64 and 2.47% lower concentration. This is due to buoyancy effect which forms upper air movement; the concentration is extracted at the occupied zone (0–1.8 m). In case WSW-1, the highest concentration was observed in ward 8109; 3.69% where most of infected cases were reported. In cases W-2 and WSW-2, where all ventilation system was not in operation, higher passive tracer concentration was observed in every contaminant-detected ward compared with other cases. In cases W-3 and WSW-3, which represented the full operation of mechanical ventilation conditions, the concentrations in ward 8104 were 4.90%, 45.4% lower than those of cases W-1 and WSW-1, respectively. In case W-3, the reduction rate of the concentration in ward 8104 was relatively low compared to case WSW-3. This is because the direct airflow from the west-facing window to the corridor was disturbed by the air supplied from the supply vent, which was located near the window and the patient. It is known that the supply of fresh air dilutes pollutants, but in the case of typical conditions, where the window is near the source, mechanical ventilation can counterbalance the reduction in pollutants.

**Table 2.** Passive tracer concentrations in wards and nurse station.

		location					
		8104	8109	8110	8111	8112	NS
case W-1T	kg′ kg^−1^ (%)	8.84 × 10^−5^ (97.7%)	1.92 × 10^−5^ (2.12%)	2.92 × 10^−5^ (7.06%)	6.39 × 10^−5^ (7.06%)	8.08 × 10^−5^ (8.93%)	3.01 × 10^−5^ (3.32%)
case W-1		8.88 × 10^−4^ (98.1%)	1.56 × 10^−5^ (1.72%)	2.62 × 10^−5^ (2.89%)	9.63 × 10^−5^ (10.7%)	1.04 × 10^−4^ (11.4%)	1.31 × 10^−5^ (1.45%)
case W-2		9.05 × 10^−4^ (100%)	4.21 × 10^−5^ (4.65%)	4.52 × 10^−5^ (4.99%)	1.22 × 10^−4^ (13.5%)	1.33 × 10^−4^ (14.7%)	1.55 × 10^−5^ (1.71)
case W-3		8.43 × 10^−4^ (93.2%)	1.48 × 10^−5^ (1.63%)	2.64 × 10^−5^ (2.91%)	6.02 × 10^−5^ (6.65%)	5.20 × 10^−5^ (5.74%)	7.12 × 10^−6^ (0.79%)
case WSW-1		6.15 × 10^−4^ (68.0%)	3.34 × 10^−5^ (3.69%)	2.07 × 10^−5^ (2.29%)	1.42 × 10^−6^ (0.16%)	6.01 × 10^−7^ (0.07%)	1.19 × 10^−8^ (0.00%)
case WSW-2		7.09 × 10^−4^ (78.3%)	4.61 × 10^−5^ (5.10%)	4.46 × 10^−5^ (4.93%)	3.25 × 10^−5^ (3.59%)	3.17 × 10^−5^ (3.50%)	5.72 × 10^−7^ (0.06%)
case WSW-3		4.77 × 10^−4^ (52.7%)	3.72 × 10^−5^ (4.12%)	3.13 × 10^−5^ (3.46%)	2.25 × 10^−6^ (0.25%)	1.54 × 10^−6^ (0.17%)	1.70 × 10^−8^ (0.00%)
attack rate		5/5	7/9	2/7	3/7	3/8	2/2

The ACH was calculated by using the concentration of the passive tracer in the ward with the passive tracer generation rate. In cases W-1, W-2 and W-3, the ACH was 21.5, 21.1 and 22.6, respectively. No significant increase in the ACH was observed by operating mechanical ventilation in ward 8104. In cases WSW-1, WSW-2 and WSW-3, ACH was 31.0, 26.9 and 40.0, respectively. ACH increased by approximately 6 when machine ventilation was operated in ward 8104. In case WSW, most of the air flowed through the side opening of the awning window to ward 8104; therefore, the air from outside did not disturb the airflow from the supply vent and more volume of air was passing through the windows compared to case W. In case W, air mostly flowed through the under part of the awning window and less air volume of air was passing through the window due to window shape and disturbed air flow from the supply vent.

## 4. Discussion

**Figure 11.**
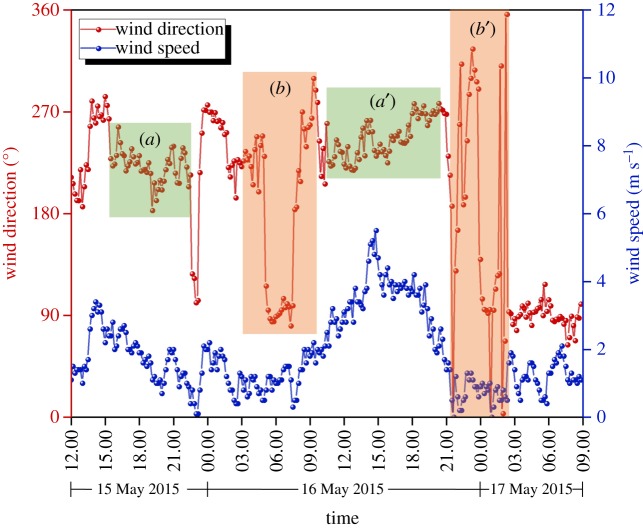
Wind speed and direction during index patient's hospitalization. (*a*), (*a*′) Consistent west, west southwest wind section; (*b*), (*b*′) East wind and random wind direction section.

The purpose of this study was to investigate the possibility of distant propagation of droplets through airflow using CFD analysis on the eighth floor of Pyeongtaek St Mary's Hospital, where the index patient was admitted. The wind speed profile was obtained and analysed based on the average wind speed values of the west, west-southwest and east winds, which were the dominant wind directions during the period in which the index patient was hospitalized. On the first day of hospitalization of the index patient (15 May 2015), the wind was mainly blowing from the west-southwest as shown in figure 11*a*, except from 22.00 to 22.30. In the first day, the wind was relatively weak, with a maximum wind speed of 3.4 m s^−1^ and an average wind speed of 1.7 m s^−1^. As shown in figure 11*b*, on 16 May, east winds were blowing from 5.00 to 7.30, and there was a possibility that the passive tracer that spread because of the west-southwest wind might re-spread to the west-facing wards because of the east wind. As shown in figure 11*a*′, winds were continually blowing from the west southwest and west until 20.00; maximum wind speed was 5.5 m s^−1^, and the average wind speed was 2.2 m s^−1^. As shown in figure 11*b*′, on the following day (16 May), the west and east winds randomly blew from 21.00 to 3.00. East wind blew with an average wind speed of 1.2 m s^−1^ and a maximum wind speed of 2.1 m s^−1^. The passive tracer that spread because of the west-southwest wind on the previous day could have re-spread to the other wards before the patient was discharged.

The passive tracer concentration in each room was analysed in terms of the door-to-door distance between wards. The passive tracer was detected in wards 8109 and 8112, which were 17.8 m apart from ward 8104, and in ward 8110, which was approximately 30 m away from ward 8104. The passive tracer concentration in ward 8109 showed the highest concentration when the west-southwest wind was blowing, indicating that MERS would have infected other patients through the airflow.

In this study, cases were simulated with steady state which cannot implement change of wind direction and speed time to time. However, one transient simulation was conducted with case W-1 to observe passive tracer spreading time. To employ realistic emission characteristics, passive tracer was set to exhale for 0.5 s with amount of 1.2 l from patient's mouth, which is considered to be one coughing time and air mass [39–41]. The passive tracer was first observed in nurse station with time of 100 s and had peak concentration at 330 s. In ward 8111 which had the highest concentration in steady simulation, passive tracer was observed in 230 s and the peak concentration was at 580 s. In ward 8110 which is the most distant ward from 8104, passive tracer was observed at 520 s and has peak concentration at 880 s. The transient simulation result tells us that in a situation when all the windows and doors are opened, the passive tracer can reach 30 m distant ward in 520 s just with one coughing scenario.

SARS is known to spread by close contact; however, Li *et al.* [14] suggested that it could possibly be an airborne infection. Li *et al.* confirmed the diffusion of contaminants through mechanical ventilation systems in the ward using CFD. In Li *et al.*'s study, the contaminant concentration was higher than that in the present study. This is attributed to the different indoor environment parameters such as air recirculation in the mechanical ventilation systems and the simulation domain size. The passive tracer concentration shown in this study might be low. However, this does not mean that the risk of infection is also low.

The concept of using passive contaminant to identify airflow rather than using particles might be different from each other. However, virus can be attached to various particle sizes and tracking of all size of particles using CFD needs extremely time-consuming process. Small size particles are more likely to travel with airflow. In the case of previous studies on SARS [14,23], indoor and outdoor airflow was analysed using passive contaminant to identify the dispersion of SARS virus based on the assumption that the droplets exhaled from the infected patient are evaporated very quickly and get small enough to flow like airflow. In this study, we adopted the concept of using passive contaminant for computing efficiency.

The first infected MERS patient was admitted to a hospital ward that had no supply or exhaust vents, indicating that the testing, adjusting and balancing (TAB) of the ventilation system was not properly carried out after the construction of the building. Especially, in the case of hospitals, it is essential to perform TAB after construction for infection control. Furthermore, it is necessary to carry out periodic TAB.

According to previous studies [38,42], natural ventilation can be used for infection control. However, in hospitals with a central corridor, such as Pyeongtaek St Mary's Hospital, there is a risk of infection being spread because of natural ventilation. For infection control, the central corridor structure should be avoided in hospitals in the design stage, and if the central corridor structure is used in hospitals, natural ventilation should be avoided and sufficient mechanical ventilation should be provided.

## 5. Conclusion

The purpose of this study was to investigate the possibility of MERS propagation through airflow on the eighth floor of Pyeongtaek St Mary's Hospital, where the index patient was hospitalized. The CFD analysis results can be summarized as follows:

— The wind data obtained for the hospitalization period of the index patient showed that the west and west-southwest winds were dominant. Therefore, the air dominantly flowed in through the west and west-southwest sides of the hospital. The passive tracer generated by the index patient in ward 8104, which is located on the west side of the building, spread towards the east-facing wards through the airflow.
— Ward 8109 showed the highest passive tracer concentration when the west-southwest wind was blowing, and mechanical ventilation was not operated, which represented the actual environmental situation during the index patient's hospitalization. The most reported MERS cases were also in ward 8109, which indicates that MERS possibly spread through the airflow.
— Real-time changes in the wind direction and wind speed were not considered in this study. However, the analysis of the wind direction and speed results showed that the passive tracer, which spread to the east-facing wards because of the west and west-southwest winds, could re-spread to the west-facing wards through the east wind.

The study results do not imply that the infection pathway of MERS is airborne. However, MERS can be infected through the airflow in specific environmental situations such as cases with inadequate ventilation of wards, cases where natural ventilation is used in central corridor structures and cases where the patient is a super-spreader.

## Data accessibility

All simulation data essential to evaluate and establish the conclusions of this study are stored in the Dryad Digital Repository: http://dx.doi.org/10.5061/dryad.4rf13r8 [43].
